# Explaining the urban–rural gradient in later fertility in Europe

**DOI:** 10.1002/psp.2720

**Published:** 2023-10-31

**Authors:** Bernhard Riederer, Éva Beaujouan

**Affiliations:** 1 Vienna Institute of Demography, Austrian Academy of Sciences, Vienna, Austria; 2 Department of Demography, University of Vienna, Vienna, Austria; 3 Department of Sociology, University of Vienna, Vienna, Austria; 4 Wittgenstein Centre for Demography and Global Human Capital (IIASA, OeAW, University of Vienna), Vienna, Austria

**Keywords:** economic structure, family norms, female education, gender roles, later fertility, population density, urban-rural differences

## Abstract

Demographic research shows that, in Europe, fertility takes place later and is lower in cities than in rural areas. One might expect fertility to be delayed in urban areas because of longer periods in education and enhanced career opportunities. We, therefore, examine how prevalent later fertility (35+ and 40+) is along the urban–rural axis, and whether differences can be explained by economic, cultural and compositional factors. We estimate multilevel random coefficient models, employing aggregated Eurostat data of 1328 Nomenclature des unités territoriales statistiques (NUTS) 3 and 270 NUTS 2 regions from 28 European countries. The urban–rural gradient in later fertility considerably diminishes once factors describing the economic environment, family and gender norms as well as population composition are accounted for. The higher prevalence of later fertility in cities is particularly associated with higher female education, greater wealth and a higher share of employment in high-technology sectors.

## 1 Introduction

Transition to adulthood has been steadily delayed in Western societies since the 1970s (Mills et al., 2011). This process contributes to later fertility, with first births to women aged 40 and older accounting for between 2% and 7% of the total first birth rate in European countries in 2018, a figure that is growing rapidly (Beaujouan & Sobotka, 2022). The prevalence of later fertility (above the age of 35 or 40) varies widely across national and subnational contexts (Leibert, 2020). The importance of national settings, in particular, countries’ economic situation, attitudes towards the family or family policies, is often emphasised to explain crosscountry differences in fertility in later reproductive life (Beaujouan & Toulemon, 2021; Sobotka et al., 2011). A more refined picture of later fertility can be obtained by understanding the aspects that account for its wide subnational variation.

Childbearing is often delayed in urban environments, and late fertility more prevalent (e.g., Buelens, 2021; Kulu et al., 2007; Riederer & Buber-Ennser, 2019. Urban and rural areas differ in many ways that may be relevant to the timing of births, such as the educational, recreational and occupational opportunities they offer, their economic structures (prevalence of knowledge work, high-tech sectors and international companies), their wealth, their lifestyle, their family cultures, their internationality and the age structure and mobility of their populations (Burtenshaw et al., 2021; Hamnett, 2021; Lichter et al., 2020; Riederer et al., 2021). Factors associated to urbanisation level may thus also be related to the prevalence of later fertility in subnational areas and its variation by degree of urbanisation. In this paper, we examine the relationship between population density and prevalence of later fertility at the subnational level, and whether this relationship is explained by differences in contextual characteristics.

After theoretically assessing why people living in urban environments may delay fertility more, we detail how a range of socioeconomic variables can be related to both later fertility and degree of urbanisation at the aggregate level. In the method part, we present the database and model used to assess this interrelationship. In the results part, we first highlight the strong relationship between cities and later fertility by using several examples of cities all across Europe. We then check whether differences between urbanisation levels persist across Europe once contextual characteristics are accounted for. The final discussion emphasises the importance of educational and professional opportunities for fertility delay across residential contexts.

## 2 Contextual Factors Relevant To Fertility Delay And Its Variation By Degree Of Urbanisation

Cities usually have lower fertility rates than suburban and rural regions in Europe (de Beer & Deerenberg, 2007; Campisi et al., 2020; Hank, 2001, 2002; Kulu, 2013; Kulu & Washbrook, 2014). According to Trovato and Grindstaff (1980), many scholars in the 1960s and 1970s thought that educational, income or employment differences between urban and rural populations were most decisive to explain the fertility contrast (compositional hypothesis). At the same time, a stronger orientation towards large families and other attitudinal and lifestyle differences pointed to the potential influence of traditional norms in rural areas (subcultural hypothesis). In the framework of the second demographic transition (SDT), Lesthaeghe (2010, p. 232) explains the fertility delay with a set of reasons that also refer to structural versus ideational factors. On the side of the former, he places mechanical factors, such as prolonged formal education and career preparation, as well as economic and social structure. On the other are cultural factors related to higher-order needs and individual autonomy, such as a greater need for self-actualisation and increased economic aspirations. As a result of these changes, men and women have different priorities in their youth before starting to think about having a family, which contributes to fewer marriages, more separations, declining fertility rates at younger reproductive ages and delayed fertility (Sobotka, 2008).

Theories emphasising the importance of gender-egalitarian norms for family formation in high-income countries complement this approach. On the societal level, fertility is lower where dominant family norms are in conflict with egalitarian gender roles and female employment (Esping-Andersen & Billari, 2015; Goldscheider et al., 2015; McDonald, 2000; Raybould & Sear, 2021). In general, female economic empowerment corresponds to both later and reduced childbearing, as it promotes women’s independence from the family (Mills et al., 2011; Osiewalska, 2018). Also, women are more likely to wait to have their first child in unsupportive societal contexts, where work and family life are difficult to reconcile and opportunity costs of childbearing are higher (Neyer, 2006). Important conditions notably entail family policies (especially affordable, high-quality childcare), flexible working arrangements and father’s involvement in the family (Matysiak & Węziak-Białowolska, 2016).

## 3 Later Fertility According To The Degree Of Urbanisation

An interesting dimension of studying later fertility across residential contexts is that several of the aspects that have contributed to its development are also attributes of city life. As developed above, childbearing delay is partly driven by increase in educational attainment, later entry into the labour force and women labour force participation in more qualified jobs. Such structural features first developed in cities and remain much more common in the urban context (Burtenshaw et al., 2021). In addition, cities are often assumed to be more progressive and less traditional than rural places (Carter & Borch, 2005; Glenn & Hill, 1977), people there embracing a lifestyle more oriented towards culture or work (Pisman et al., 2011; Riederer & Buber-Ennser, 2018). Thus, metropolitan areas are often emphasised as the forerunners of the SDT, where attitudes and norms towards the family are more flexible and alternative couple and fertility patterns develop quickly, whereas traditional family views and behaviours remain longer predominant in rural areas (Lesthaeghe, 2010; Valkonen et al., 2008; Walford & Kurek, 2016). Alternative family forms that characterise the SDT, such as unmarried cohabitation, short childless unions and separation, are highly relevant for later fertility as they contribute to (re)starting childbearing at a later age (see, e.g., Thomson et al., 2012). During the last decades, urban-rural gradients in SDT-related attitudes and behaviours may have been attenuated due to ongoing secularisation, globalisation, technological change and the spread of (new) mass media, resulting in mixed empirical evidence (Lesthaeghe & Neels, 2002; Valkonen et al., 2008).

Although research often refers to urban–rural contrasts, there is rather an urban–rural continuum, consisting of a broad spectrum of intermediate levels of urbanisation, with corresponding differences in residential environment and in fertility behaviour. Within a country, fertility is particularly late in the metropolitan regions that include the country’s capital; other types of less densely populated urban areas, such as smaller cities and local industrial centres, generally postpone fertility less; and rural areas have the earliest birth schedules (Buelens, 2021). Metropolitan areas themselves are diverse: fertility rates are higher in suburban areas (Kulu & Washbrook, 2014). First, they offer a better environment for a family, such as larger housing with garden and green areas (Vobecká & Piguet, 2012). Often, people move there to start a family in an environment that is better suited to their children’s needs and activities, while still being close to the opportunities the city has to offer (Kulu & Washbrook, 2014). Their values do not seem to differ much from those of the city dwellers (Pisman et al., 2011). Second, people in low-paid jobs for whom the city is unaffordable tend to live in (very different) suburban areas, marking strong suburban inequalities (Bailey & Minton, 2018; Weck et al., 2023). This group, often comprised of migrants from non-European origin and people from modest background, generally has more children than the average so that their last children are often born at older maternal ages (Kulu et al., 2017; Toulemon, 2004). Overall, we expect a fairly linear variation in later fertility across the urbanisation gradient, with large shares of late fertility in very high-density areas.

We also note different levels of urbanisation between urban and rural areas within and between European countries (European Environment Agency, 2009), as well as very different prevalence of late fertility (Buelens, 2021). Population density is particularly low in the Nordic and Baltic countries while it is the highest along the diagonal from the United Kingdom to Italy. The number of urban areas also varies considerably between countries. In this paper, we use population density to represent the range of urbanisation levels, with the drawback that density is not exactly the same in urban and rural areas of different countries. Later fertility, represented as the contribution of women aged 35+ (or 40+) to the total fertility rate (TFR), is also subject to national as well as international variations. It is much more prevalent in the South of Europe, and much less so in countries of Central and Eastern Europe (Beaujouan, 2020). These limitations are partly dealt with in our models, as detailed later.

## 4 Factors Of Later Fertility Differentials Along The Urban–Rural Continuum

In this section, we explore which contextual factors may underlie the variation in later fertility along the urban–rural continuum, highlighting the importance of socioeconomic, cultural and compositional factors. Such elements are hardly independent of each other (Riederer & Buber-Ennser, 2019). For instance, highly educated women are more likely to live in cities where they pursue professional careers, and tend to have less traditional attitudes and lifestyles. The urban environment, including the educational and economic opportunities and the corresponding lifestyles, also leads to a different composition of the population than in small towns and rural areas. Despite difficulties in disentangling their effects, the analytical distinction between these elements has been fruitful in identifying reasons for differences in fertility levels by degree of urbanisation (see Campisi et al., 2020; Hank, 2002; Kulu & Washbrook, 2014; Riederer & Buber-Ennser, 2019). In this section, we discuss the potential role of the socioeconomic environment, family and gender norms and population composition for differences in fertility timing along the urban–rural continuum.

### 4.1 Level of education

The role of education for delay and later recovery of childbearing is emphasised in research on regional fertility variations. Indeed, highly educated women are more likely to have children at later ages, when less educated women have already completed their family (Neels et al., 2012). Findings of Kulu et al. (2007) for Sweden indicate that ‘the higher fertility of older women in the large cities is mostly the result of the larger proportion of highly educated women‘ (Kulu et al., 2007, p. 277). This is in line with findings of Riederer and Buber-Ennser (2019) who report that postponing fertility intentions is more common in urban than in rural regions in Europe, likely owing to the larger proportion of highly educated women. Michielin (2004) even supposed that urban fertility is mainly driven by female education as the highly educated may be ‘more oriented to urban ways of life’ (Michielin, 2004, p. 343). Kulu et al. (2007, p. 279) also refer to a ‘preferred lifestyle’ in urban settlements, but discuss in addition other mechanisms possibly leading to later fertility, such as high competition on urban labour markets.

### 4.2 Economic environment

Several studies suggest that economic factors are at least partly responsible for urban-rural fertility differentials (Campisi et al., 2020). Kulu and Washbrook (2014, p. 169f.), for instance, state that living costs are higher in cities (e.g., housing), that children are more expensive and time-consuming for parents (e.g., after-school activities, extracurricular activities, availability of various shops and attractions), that opportunity costs of childbearing are higher (more opportunities for work and leisure), and that life is more competitive in large cities than small towns and rural regions. In this context, higher living costs and economic competitiveness alone may contribute to later births if young people postpone starting a family until they are financially secure (Kulu, 2013; Kulu et al., 2007). In addition, European cities are characterised by professionalisation trends (Hamnett, 2021; Riederer et al., 2021), offering more opportunities and jobs in tertiary education, business, science and technology, sectors that require a high initial career and time investment and may contribute to lower fertility at younger ages (Kravdal, 1994). These trends often go hand in hand with better childcare infrastructure, making it easier to combine a career with motherhood later in life (Riederer & Buber-Ennser, 2018). Although economic conditions in metropolitan areas vary, the urban economic environment seems to encourage later fertility.

### 4.3 Family and gender norms

More liberal norms towards family and acceptance of women’s work may lead to more favourable conditions for fertility at a later age. Specifically, broad acceptance of maternal employment and respective policy support (particularly, affordable and good quality childcare) are understood as a prerequisite for combining a career and motherhood in modern societies (Esping-Andersen, 2009; Goldscheider et al., 2015; Matysiak & Węziak-Białowolska, 2016). Women’s work is more accepted in cities, and the necessary childcare facilities have developed more quickly across urban than rural areas in the last decades (Wood & Neels, 2019). Hence, in urban regions with a higher prevalence of female employment, circumstances may be more favourable to fertility recovery at later ages.

Flexible family norms, by allowing childbearing in diverse family circumstances, may also facilitate fertility at later ages. In particular, research on the United States (Fuguitt et al., 1989; Snyder et al., 2004) describes urban–rural fertility differentials as a consequence of differences in marital status and of the lower age at first marriage in rural areas (cf. Vobecká and Piguet, 2012, p. 226). Higher divorce rates indicate more liberal family norms, and divorce rates tend to be higher in European cities (e.g., Gautier et al., 2009; Lyngstad & Jalovaara, 2010). The repartnering of divorcees may then contribute to later fertility in urban areas (Thomson et al., 2012).

### 4.4 Population composition

The composition of the population varies systematically along the urban–rural continuum and has likely an impact on later fertility. The age structure also affects the prevalence of births by women age 35+, and we explain below how we account for this in the construction of our dependent variable. Rural regions in the intertwined processes of depopulation and population ageing are often characterised by out-migration, lower economic development and problems in the provision of social services; including childcare services and schools (Leibert & Golinski, 2017; Reynaud & Miccoli, 2018). Given out-migration due to economic uncertainty, a lack of future prospects and missing possibilities to combine work and family, later fertility is probably less relevant in such settings.

Migration particularly matters for regional fertility differentials (e.g., Kulu, 2006; Michielin, 2004; Vobecká & Piguet, 2012). Selective migration to cities may contribute to later fertility, as metropolitan regions attract highly qualified natives and foreigners who are more likely to postpone childbearing (e.g., Riederer, 2021). In addition, international immigrants are generally more often found in urban than in rural areas. Metropolitan areas and cities have larger share of migrants (Lichter et al., 2020), who have larger family size (Kulu et al., 2017), which may contribute to later fertility. Indeed, while they may begin to postpone childbearing as part of an adjustment process, their fertility may remain temporarily high at older ages, thereby reinforcing the differentials in later fertility along the urban–rural continuum.

In summary, urban areas—and particularly international cities and metropolitan regions—in Europe are characterised by higher levels of later fertility than rural areas. We hypothesise that characteristics of the economic environment, family and gender norms and population composition account for differences along the urban–rural continuum. Finally, we expect our hypotheses to hold (broadly) across different national contexts.

## 5 Data And Methods

### 5.1 Construction of indicators

Our dependent variable is an indicator of later fertility at the subnational level across European countries. As the relevance of later fertility obviously depends on the number (and share) of women of the respective age group in a region, we use age-specific fertility rates (ASFR) instead of number of births to calculate late fertility prevalence. Hence, our indicator corresponds to the contribution of women aged 35+ (or 40+) at birth to the TFR per calendar year.^1^

Variation of the late fertility indicator is observed along the urban–rural continuum. Going beyond the urban–rural dichotomy, the urban–rural continuum (i.e., the degree of urbanisation) is indicated by population density (persons per square kilometre). At the nomenclature des unités territoriales statistiques (NUTS) 3 level, the population density variable represents urbanisation along the gradient from very dense cities to rural areas through less densely populated urban areas (e.g., suburbs and towns). Using population density at NUTS 3 level makes it possible to clearly distinguish between metropolitan, urban and rural areas (Campisi et al., 2020), and to identify specific areas in many metropolitan regions (e.g., Athens, Greater London, Île-de-France). Our analyses are, nonetheless, also conducted at NUTS 2 level. First, the geographic detail of NUTS 3 regions differs across European countries, whereas region sizes do not vary that strongly at NUTS 2 level. Second, some of the other covariates (see below) are only available at NUTS 2 level. In short, NUTS 3 level analyses allow for a more accurate and fine-grained picture and NUTS 2 level analyses may be preferable in terms of comparability across indicators and countries.

Other covariates indicate aspects of education, the economic environment, family and gender norms and population composition. *Education* is measured by the share of women with tertiary education among all women aged 25–64. The *economic and educational environment* in each area is captured by gross domestic product per capita (in purchasing power standards and as percentage of the European Union average) and the share of the high-tech sector in the economy (i.e., employment in high-technology manufacturing and knowledge-intensive high-technology services). Indicators of dominant *family and gender norms* comprise the share of divorced persons (including persons whose registered partnership was legally dissolved) in the population and the ratio of male-to-female employment (age 25–54). Finally, the *composition of the population* is described by the share of the population aged 60 and over among the total population and the share of foreign-born women among female population (age 25–54). Most measures refer to the year 2018.^2^ Exceptions are figures for Germany that refer to 2017 and figures for the share of divorced persons that have been calculated on the basis of the population censuses in 2011.^3^ The aggregated data has been retrieved from various EUROSTAT databases (for details, see Supporting Information: Table A.1).

### 5.2 Analytical strategy

Our analytical strategy comprises several steps. First, we compare the share of ASFR 35+ and 40+ in total fertility in major cities to the respective country average in a descriptive analysis. Exemplary presentations of the results for Germany and the United Kingdom provide a more detailed picture of regional differences. Second, we employ multilevel models with varying model specifications. For multilevel analyses, we use aggregated data for 1328 NUTS 3 and 270 NUTS 2 regions from 28 European countries.^4^

Although Campisi et al. (2020) argue in favour of spatial models to analyse regional variations in fertility, we decided to use multilevel mixed-effects models (i.e., models with both random intercepts and random slopes). First, we want to avoid overlooking cross-country heterogeneity in urban–rural differences (i.e., the magnitude of coefficients). Differences between regions are larger in some countries than in others for manifold reasons (e.g., the process of defining NUTS regions, the degree of urbanisation of a country, geographical structure) and this affects estimated associations. Erroneously assuming invariant coefficients across countries reduces the estimation precision (Heisig et al., 2017) and can lead to serious biases even if the fixed effects are specified correctly (Schmidt-Catran & Fairbrother, 2016). The estimation of random slopes avoids such problems. Second, we focus on the role of regional characteristics for fertility differences in the urban and rural contexts, explicitly assuming differences by degree of urbanisation due to characteristics of cities and the urban context that go beyond geographical proximity. Given our research interest, the correct specification and interpretation of spatial effects are not straightforward (see Golgher & Voss, 2016).^5^ As covariates and the correct estimation of random slopes, rather than spatial effects per se, are central to our main research question, we prefer multilevel mixed-effects models to spatial models in our specific case. Nevertheless, we present sensitivity analyses in the Supporting Information: Appendix to check for spatial autocorrelation in multilevel models (computing Moran’s I with residuals) and whether results in standard spatial models are similar to those in multilevel mixed-effects models.

A series of three-level mixed-effect models with random intercepts and slopes serves to analyse the association between population density (indicating the degree of urbanisation) and later fertility on NUTS 3 level. In the first model, we include only a fixed effect of population density. In the second model, we additionally introduce a random effect on the country level to explore how the coefficient varies over countries (random slope). In the third model, we additionally include context variables available at NUTS 3 level (fixed and random effects). The fourth model also contains context variables at NUTS 2 level (only fixed effects due to low case numbers). This final model can be expressed as:

$$y_{ijc}=\alpha +\zeta_{jc}+\zeta_{c}+\left(\beta_{1}+\zeta_{1c}\right)x_{ijc}+\beta_{2}x_{jc}+\varepsilon_{ijc},$$

$$y_{ijc}=\left(\alpha +\beta_{1}x_{ijc}+\beta_{2}x_{jc}\right)+\left(\zeta_{jc}+\zeta_{c}+\zeta_{1c}x_{ijc}+\varepsilon_{ijc}\right),$$

where *y*_ijc_ is the dependent variable ‘later fertility’, *α, ζ*_jc_ and *ζ*_c_ are intercept(s), with *ζ*_jc_ varying at NUTS 2 level and *ζ*_c_ varying at country level (random intercepts); *x*_ijc_ and *x*_jc_ represent explanatory variables at NUTS 3 and NUTS 2 level, respectively; vector *β*_1_ includes the regression slope coefficients of NUTS 3 level variables and vector *β*_2_ the regression slope coefficients of NUTS 2 level variables; *ζ*_1c_ allows the regression coefficients of NUTS 3 level variables to vary across countries (random slope). Summarising (*α* + *β*_1_ *x*_ijc_ + *β*_2_ *x*_jc_) is the fixed part and (*ζ*_jc_ + *ζ*_c_ + *ζ*_1c_ *x*_ijc_ + *ε*_ijc_) is the random part of the model. Most importantly, random intercepts account for different levels of later fertility by country and NUTS 2 region, while random slopes reflect cross-country heterogeneity regarding all NUTS 3 level covariates, including urban–rural differences in later fertility. The estimated relationship between later fertility (*y*) and population density (*d*) can thus be described as (*β*_pd_ + *ζ*_pdc_) *d*_ijc_ in:

$$y_{\text{ijc}}=\alpha +\zeta_{\text{jc}}+\zeta_{\text{c}}+\left(\beta_{\text{d}}+\zeta_{\text{dc}}\right)d_{\text{ijc}}+\left(\beta_{1}+\zeta_{1\text{c}}\right){\text{x}}_{\text{ijc}}+\beta_{2}{\text{x}}_{\text{jc}}+\varepsilon_{\text{ijc}},$$

where (*β*_d_ + ζ_dc_) describes the regression slope coefficients of population density (*d*_ijc_) that vary across countries (*ζ*_dc_), whereas *β*_1_, *ζ*_1c_ and *x*_ijc_ refer to other NUTS 3 level covariates.

Afterwards, we use a series of two-level models to refine which groups of context variables explain the association between population density and later fertility. We conduct this analysis on NUTS 2 level because of better comparability of NUTS 2 regions across European countries and because not all necessary context information is available on NUTS 3 level. As the number of observations is much smaller at NUTS 2 level, these models include a random slope only for population density. We estimate models separately considering indicators of female education, economic environment, family and gender norms and population composition, before including all of them in a final model. In sensitivity analyses (shown in the Supporting Information: Appendix), we follow the same procedure with different model specifications (including spatial models) to demonstrate the robustness of our findings and to test changes in the coefficients of population density across models.

### 5.3 Choice of standardisation method

All described models are run for both the share of ASFR contributed by women aged 35+ and by women aged 40+ to TFR. In addition, standardisation is meaningful to make coefficient sizes comparable. We test two different methods of standardisation to the sample mean, using the country-specific standard deviation (SD) in one case and the total sample’s SD in the other.^6^ Within-country standardisation is valuable in multilevel settings (e.g., Horn, 2007): it accounts for differing degrees of variation within countries and avoids standardised coefficients above 1. Regions are thus compared to other regions within their country and coefficients indicate an increase in one SD within a country instead of one SD across all regions.

We identify the most accurate method of standardisation here and then focus on models employing the preferred method throughout the rest of the article. Table 1 gives the results of our multilevel models on NUTS 3 level applying both ways of standardisation, using the grand mean and within-country SD (M1–M4, left columns)^7^ or the grand mean and the total sample’s SD (N1–N4, right columns). Overall, all models show a positive association between population density and later fertility: the higher the population density, the larger the share of ASFR 35+ (or 40+) in TFR in a region on average. As expected, coefficients of population density are larger in the models without covariates (M1–M2, N1–N2) and become smaller when context characteristics are included (M3–M4, N3–N4). Akaike information criterion and Bayesian information criterion improve with every step, justifying the decision to account for between-country variation and to add further covariates (Tables 2 and 3).

Models M1 and N1 only include country-fixed effects. In models M2 and N2, the coefficient of population density is allowed to vary across countries (country random effect). Although SDs of 0.18 (panel A) and 0.21 (panel B) indicate considerable variation between countries, the coefficient of population density in model M2 is barely different from that in model M1 (0.53). However, coefficients for population density in models N1 and N2 dramatically increase from 0.40 to 0.71 (panel A) and from 0.46 to 0.70 (panel B). In addition, SDs in model N2 are larger than 0.40, which indicates that standardised coefficients for some countries are larger than 1 and that we overestimate the strength of the association if using the total sample’s standardisation. The same conclusions can be drawn from models at NUTS 2 level where coefficients in model Y2 exceed values of 1 (1.13 in panel A and 1.18 in panel B of Supporting Information: Table A.3).

## 6 Descriptive Results: Major Cities And Later Fertility

**Figure 1:**
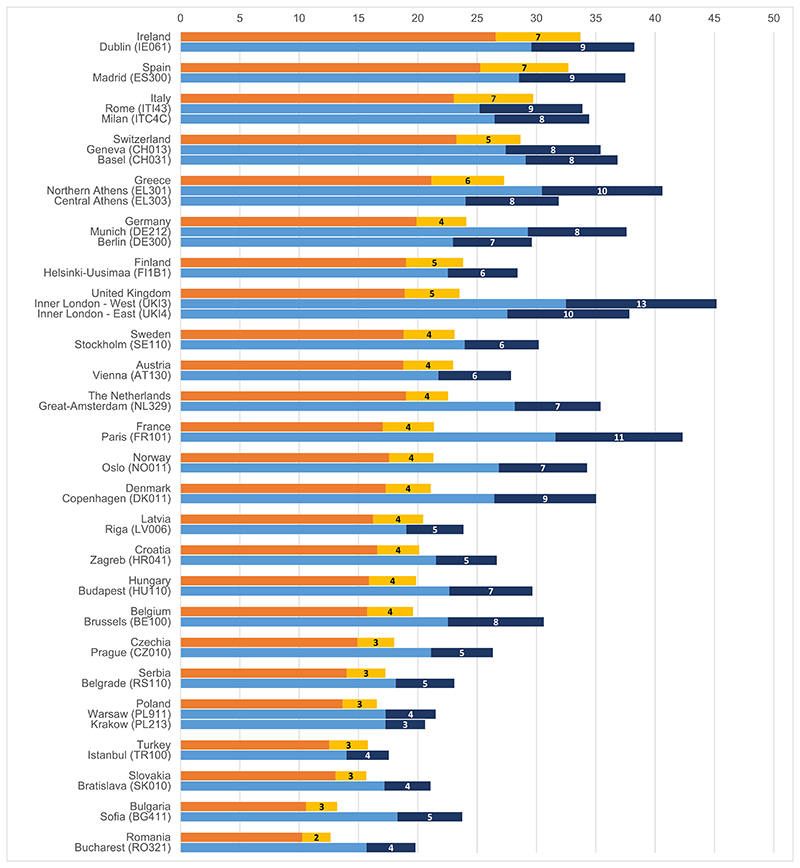
Share of age-specific fertility rates 35–39 and 40+ in total fertility rate in Europe (in %). *Source*: Eurostat (2021); figures refer to 2018, except for Germany (2017); own figure.

In all the European countries considered, later fertility appears to be more prominent in the capital cities and other large cities than in the national average (Figure 1). The share of fertility by women 35+ in total fertility is, for instance, larger in Paris than in France, larger in Berlin or Munich than in Germany, larger in Praha than in Czechia, larger in Athens (especially in the North) than in Greece, or larger in (particularly Inner) London than in the United Kingdom on average. The shares of later fertility differ considerably by countries and cities—Inner London West is characterised by the highest share of births at ages 35+ (45%) and births at ages 40+ (13%), while Romania is the country with the lowest share of ASFR 35+ in total fertility (slightly above 17%)—but the general citycountry contrast is very consistent.

**Figure 2:**
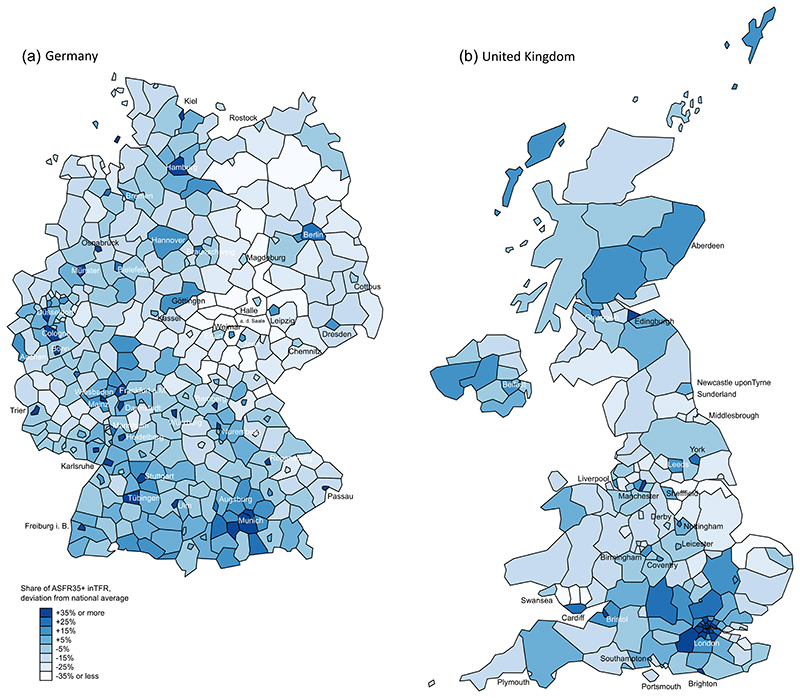
Share of age-specific fertility rates 35+ in total fertility rate by NUTS 3 region for (a) Germany and (b) the United Kingdom, deviation from the respective national average. *Source:* Eurostat (2021); figures refer to 2017 (Germany) and 2018 (United Kingdom); own figure.

The examples of Germany and the United Kingdom provide a more complete picture of the regional variation in later fertility. The darker patches in Figure 2 show regions with a higher share of later fertility (ASFR 35+) in total fertility, compared to the respective national average. Later fertility tends to be more common in metropolitan areas and large cities. Prominent examples are Cologne, Hamburg or Munich in Germany and London or Manchester in the United Kingdom. In addition, even cities with lower levels of later fertility often have higher levels than surrounding areas. This is the case for eastern German cities such as Leipzig or Dresden, and for Liverpool or Glasgow in the United Kingdom. The high level of geographical detail of NUTS 3 divisions allows for additional insights: In Germany, many small commercial or university towns such as Bamberg, Heidelberg, Karlsruhe or Passau are also characterised by high prevalence of later fertility. The representation of Greater London reveals differences within metropolitan regions: inner London areas like Wandsworth (48%), Lambeth (45%) or Camden and the City of London (44%) have much higher shares of late fertility than outer London areas like Croydon (26%), Harrow and Hillingdon (25%) or Barking and Dagenham and Havering (23%).

Although the general pattern of higher late fertility in cities holds across Europe and can be observed at both NUTS 3 and NUTS 2 levels (see Supporting Information: Figures A.1 and A.2), there are also exceptions. These exceptions are usually workingclass cities with a mining and manufacturing heritage, like Gelsenkirchen in the German Ruhr Area or Katowice in Poland, or harbour cities, like Middlesbrough in the United Kingdom or Naples in Italy. Nevertheless, these exceptions do not contradict our general assumption. Rather, they point to the relevance of the economic context for urban–rural differences in later fertility.

## 7 Results From Multilevel Models: Degree Of Urbanisation And Later Fertility

To explore how much the association between population density and later fertility can be explained by education, economic context, family and gender norms and population composition, we conducted a series of models with all variables measured at NUTS 2 level (Table 2). The results for the share of ASFR 35+ (panel A) and 40+ (panel B) are not substantially different and lead to the same conclusions. The bivariate association between population density and later fertility (models X1 and X2) is remarkable. The values of the coefficients *b* range between 0.55 and 0.65 and are of a comparable magnitude to those obtained with measures at NUTS 3 level (compare Table 1, M1 and M2). Further, in line with the results at NUTS 3 level, SD_c_ of 0.28 and 0.29 (model X2) indicate considerable variation in the association between countries.

Our findings indicate that regional variations in education are important for differences in later fertility along the urban–rural continuum. The coefficients indicating the association between population density and later fertility (b) are substantially smaller when education (share of women with tertiary education) is included into the model (model X3 in Table 2). The coefficients shrink from 0.55 to 0.30 (ASFR 35+) and from 0.62 to 0.39 (ASFR 40+), respectively. The reduction in coefficients is even larger when indicators of economic environment are included instead of education (0.16 and 0.29 in model X4). As expected, a higher share of highly educated women, a higher average gross domestic product (GDP) and a higher share of the high-tech sector are all associated with a higher share of late fertility. Comparing the coefficient of population density between models in which each of the three variables is entered separately (Table 3), the magnitude of the reduction in the coefficients seems comparable (coefficients between 0.26 and 0.30). When indicators of family and gender norms are included (model X5), there is no change in the coefficient of population density (Table 2). Some changes, albeit smaller than those in models considering the educational or economic environment, are found when indicators of population composition are included (coefficients of 0.45 and 0.54 in model X6, respectively). These findings are also consistent across the other model specifications, and tests of differences between coefficients support our interpretations (Supporting Information: Table A.4).

Finally, both the coefficient of population density b and its variation between countries SDc are reduced most when all variables are entered simultaneously in model X7 (Table 2). The coefficient now amounts to 0.12 (compared to 0.53 in model X2) and 0.23 (compared to 0.62 in model X2); the SDc to 0.15 (compared to 0.28 in model X2) and 0.18 (compared to 0.29 in model X2). Altogether, our analyses suggest that regional characteristics, and in particular the educational and economic environment, explain a very substantial part of the relationship between the degree of urbanisation and later fertility on NUTS 2 level and its variation across countries.

## 8 Further Findings And Sensitivity Analyses

Other findings stand out as well. First, we observe significant associations of the indicators of gender and family norms and population composition with indicators of later fertility (Table 2): The lower the share of divorcees, the higher the share of ASFR 35+ in total fertility (models X5 and X7), and the higher the male-to-female employment ratio, the higher the share of ASFR 35+ and 40+ in total fertility (model X7). The higher the share of population age 60+, the lower the share of ASFR 35+ and 40+ (model X6). This association disappears, when all covariates are introduced (model X7), indicating that the association is mostly driven by the difference in the socioeconomic context of ageing areas. The higher the share of the foreign-born female population, the higher the share of ASFR 35+ and 40+, but the magnitude of the coefficient is small (models X6 and X7).

Second, results of multilevel models with population density and later fertility measured at NUTS 3 level are very consistent with results obtained at NUTS 2 level. The coefficient for population density and its decrease when contextual covariates are added are of comparable magnitude, and associations between later fertility and contextual covariates hardly differ from those obtained in models at NUTS 2 level (Supporting Information: Tables A.5 and A.6). Nevertheless, two deviations have also to be noted. In NUTS 3 level models, only the contextual characteristics measured at NUTS 3 level explain a substantial part of the positive association between population density and later fertility. The coefficient of population density hardly changes when contextual characteristics only available at NUTS 2 level are additionally entered (compare M3 and M4 in Table 1). This suggests a high relevance of measuring all covariates at the same level to avoid inaccuracies.

In addition, the higher the share of divorcees at NUTS 3 level, the higher the share of ASFR 40+ in total fertility at NUTS 3 level (Supporting Information: Table A.5). Seemingly, this is in contrast to the NUTS 2 level analysis (negative effect on the share of ASFR 35+). The variation in the coefficient across countries SD_c_, however, is larger than the estimated coefficient *b*. On NUTS 3 level, estimated associations are positive for most countries (e.g., France, Belgium), close to zero for some (e.g., Poland, Czechia), and even negative for others (in particular for Italy and the United Kingdom). When we add a random slope for the share of divorces on NUTS 2 level, the variation in the coefficient across countries is also larger than the estimated coefficient. Estimated associations are again negative for Italy and the United Kingdom and positive for France. Both, negative effects due to separations as well as positive effects due to repartnering may be at work (Thomson et al., 2012).

Finally, in models both at NUTS 2 and NUTS 3 levels, female education is by far the most important predictor of later fertility. In models at NUTS 3 level, NUTS 3 level measures of population density and GDP show coefficients of similar magnitude as female education, but female education is the only NUTS 2 level measure with a strong association with later fertility at NUTS 3 level. Female education is thus confirmed to be the most important predictor for later fertility (e.g., Compans, 2021; Neels et al., 2017).

We conduct analyses to check whether spatial autocorrelation could seriously bias our results. Computations of Moran’s *I* show that there is huge spatial dependence in later fertility (values ranging from 0.75 to 0.81). However, most spatial dependence is accounted for in our multilevel models: In models at NUTS 3 level, remaining spatial dependence indicated by Moran’s *I* on residuals is close to zero (between 0.01 and 0.03). In models at NUTS 2 level, Moran’s *I* ranges from 0.11 to 0.30 (Supporting Information: Table A.7). In addition, conclusions obtained with spatial autoregressive models are the same as with other model specifications. When all the covariates are introduced, the coefficients for population density are almost identical (Supporting Information: Table A.8).

## 9 Summary And Discussion

Previous research consistently shows that European cities and urban regions are characterised by lower fertility than rural regions (e.g., Hank, 2001; Kulu & Washbrook, 2014). Some studies explicitly address postponement of childbearing in cities (e.g., Kulu et al., 2007; Riederer & Buber-Ennser, 2019), but differences in later fertility across the urban–rural continuum and its factors have not been examined. Addressing this research gap, we analysed differences in later fertility in Europe by degree of urbanisation, using aggregated Eurostat data of 1328 NUTS 3 and 270 NUTS 2 regions from 28 countries.

First, our findings confirmed that later fertility is much more common in cities than on average. Rare exceptions are some working-class cities. Second, results from multilevel random coefficient models indicated a remarkable link between population density and later fertility with some variation in effect size across countries. Third, stepwise model building suggested that the association between population density and later fertility can be largely explained by the share of highly educated women and the economic environment.

Methodologically, our multilevel mixed-effect models demonstrated that it is important to consider not only random intercepts but also random slopes. The strength of the association between population density and the share of later fertility in total fertility varied significantly across countries (as did the associations with other contextual characteristics). We also showed that caution is needed in assessing the effect sizes of contextual variables, as only standardisation using within-country SDs allowed to compare countries.

The particular relevance of the educational and economic environment for later fertility in general, and for the association between population density and later fertility in particular, supports the claim that occupational opportunities in urban environments are highly relevant for urban–rural differentials in childbearing postponement (e.g., Riederer & Buber-Ennser, 2019). Although gender and family norms as well as population composition also matter for later fertility, they seem to be less relevant for differentials in later fertility across the urban–rural continuum. In line with the demographic literature, the tertiarisation of urban economies seems to be most important for urban-rural differences in (later) fertility (e.g., Michielin, 2004; Vobecká & Piguet, 2012).

The high relevance of the economic context for urban-rural differences in later fertility reinforces also the picture of a divide between the city and less densely populated, ageing areas, where economic differences may also exacerbate other behaviours. Together with findings on education, it furthermore points to issues of work-family compatibility. In the wake of women’s empowerment, the tertiarisation of urban economies and skill-biased technological change, women’s educational and economic aspirations and opportunities often compete with bearing and raising children. Particularly in cities, where later fertility is likely to be more prevalent, policies that promote work-family reconciliation through increased gender equality and the availability of quality childcare are crucial (Matysiak & Węziak-Białowolska, 2016).

Our indicators of family and gender norms did not affect the association between population density and later fertility. Either family and gender norms are little active in explaining the urban–rural gradient or better measures of family and gender norms are needed. The huge variation of associations between the share of divorcees and later fertility across countries suggest that effects of divorce on fertility may depend on the country-specific institutional context. Furthermore, our results were not consistent with usual expectations: the more conservative family and gender norms were (indicated by lower divorce rate, higher male-to-female employment ratio), the higher later fertility was. This makes more sense, when we think about large families and higher-order births than about ‘postponement’ of the first birth.

The primary limitation of our study is that we could not distinguish between later births indicating ‘postponement’ of the first birth and those indicating family enlargement by mothers. Despite the current development of a larger variety of fertility indicators at NUTS 2 and NUTS 3 levels (Nisén et al., 2021), the coverage remains limited to countries where large data sets and notably registers are made available for such calculation. Future studies should draw a more detailed picture that draws on other indicators such as the share of women remaining childless. Indeed, though this has not (yet) been found at the cross-country level in the past generations, one can expect that with the increase in age at first birth, the share of women having children later and of women remaining childless will become increasingly correlated, and this would be better observed at a more refined geographical scale.

Unfortunately, information on some context characteristics were only available at NUTS 2 level. The NUTS 2 scale may not be refined enough to seize the subtility of local variations, which is consistent with the fact that public service infrastructure is constructed at a very local scale. For other characteristics, information was not even available at NUTS 2 level. For instance, we did not find indicators referring to environmental quality (e.g., air quality, availability of green spaces), housing (e.g., crowded housing, housing costs) and other aspects of quality of life (e.g., childcare, crime rates, living costs, poverty, public transport) for enough regions to include them in our analyses. They are all parts of the context in which childbearing decisions are made, realised, postponed or abandoned. Information at a more refined geographical scale would also allow to dig into the complexity of metropolitan areas, and future studies of fertility quantum and timing could focus on the heterogeneity of the populations living in urban areas. Finally, modelling strategies that reflect our understanding that urban contextual factors affect the surrounding areas (but not vice versa) and that simultaneously integrate multilevel mixed-effects models and spatial models to avoid that random slopes at the country level are affected by spatial autocorrelation, would be promising.

## 10 Conclusion

Our findings primarily point to the importance of the educational and economic context in shaping alternative life goals and opportunity costs of childbearing. The relationship of elements such as lifestyle, quality of life and wealth to the timing of childbearing can be further explored, as they still tend to vary across urban–rural contexts and countries (Shucksmith et al., 2009; Sørensen, 2014). The literature indicates that early parenthood may have negative consequences for parents’ subjective well-being, while the evidence on later fertility remains inconclusive (Kohler et al., 2005; Mirowsky & Ross, 2002; Riederer, 2018). Our main message that context matters is very much in line with the research on later fertility. As Beaujouan and Toulemon (2021, p. 13) recently found at the country level, contextual effects can dominate individual constraints (including biological ones) and are often decisive for the occurrence of later births. Cities as economic centres of knowledge societies with important high-tech sectors, characterised by international competition, digitalisation and a high dependence on high education, will probably remain distinct from rural areas in the future. Their characteristics appear to be particularly relevant to later childbearing and are likely to shape future demographic trends and behaviours.

## Supplementary Material

Supplementary material

## Figures and Tables

**Table 1:** Associations between population density and later fertility on **NUTS** 3 level according to standardisation method (multilevel mixed regression models)

Model	Standardisation with grand mean and within-country standard deviation				Standardisation with grand mean and standard deviation across all NUTS 3 or NUTS 2 regions			
	M1	M2	M3	M4	N1	N2	N3	N4
A. Share of ASFR 35+ in TFR								
Population density (b)	0.52***	0.53***	0.26***	0.24***	0.40***	0.71***	0.26***	0 21***
SD_C_ population density	-	0.18	0.09	0.06	-	0.43	0.09	0.07
AIC	3171	3142	2840	2808	2211	2112	1776	1726
BIC	3197	3173	2902	2891	2237	2143	1839	1809
LR *χ*^2^ test against previous model	-	30.7***	344.9***	40.2***	-	101.3***	347.1***	58.4***
B. Share of ASFR 40+ in TFR								
Population density (*b*)	0.53***	0.53***	0.30***	0.28***	0.46***	0.70***	0.32***	0.28***
SD_C_ population density	-	0.21	0.14	0.11	-	0.42	0.11	0.07
AIC	3189	3141	2949	2914	2150	2060	1853	1802
BIC	3215	3172	3011	2997	2176	2091	1915	1885
LR *χ*^2^ test against previous model	-	50.5***	203.5***	43.4***	-	91.9***	219.0***	58.7***
Included in model								
NUTS 3 covariates	-	-	Incl.	Incl.	-	-	Incl.	Incl.
NUTS 2 covariates	-	-	-	Incl.	-	-	-	Incl.

*Note*: *N*_country_ = 28; *N*_NUTS 2_ = 270; *N*_NUTS 3_ = 1328. Population density at NUTS 3 level. NUTS 3 covariates: GDP/capita (%), share of divorced persons (%; in 2011), share of couples with four or more children (%; in 2011), share of population age 60+. NUTS 2 covariates: share of high-tech sector (%), share of women with tertiary education (%), share of foreign-born female population (%; age 25–54), ratio of male-to-female employment (ratio of %, age 25–54). b, regression coefficients. SD_C_, random-effect on country level (i.e., variation of coefficient b across countries). Abbreviations: AIC, Akaike information criterion; ASFR, age-specific fertility rates; BIC, Bayesian information criterion; GDP, gross domestic product; LR, likelihood ratio; TFR, total fertility rate.

*** p≤ 0.001

**Table 2:** Associations between population density and later fertility on NUTS 2 level (multilevel mixed regression models), standardisation with grand mean and within-country standard deviation.

Model	X1	X2	X3	X4	X5	X6	X7
	b	b	b	b	b	b	b
A. Share of ASFR 35+ in TFR							
Population density	0.55***	0.55***	0.30***	0.16*	0.58***	0.45***	0.12*
SD_c_ Population density		0.28	0.19	0.19	0.28	0.26	0.15
Education							
Share of women with tertiary education			0.49***				0.41***
Economic environment							
GDP/capita (%)				0.27***			0.20***
Share of high-tech sector				0.37***			0.17**
Family and gender norms							
Share of divorced persons (2011)					-0.09****		-0.14***
Ratio of male-to-female employment (age 25–54)					-0.03		0.16***
Population composition							
Share of population age 60+						-0.15**	0.00
Share of foreign-born female population (age 25–54)						0.04***	0.03**
Intercept	1.48**	0.80*	0.35	0.18	0.83*	0.89**	0.54
SD_c_ intercept	2.84	1.87	1.80	1.68	1.87	1.54	1.44
SD residual	0.76	0.72	0.61	0.61	0.72	0.71	0.55
AIC	750	729	644	640	729	715	590
BIC	764	747	666	665	755	741	633
LR χ^2^ test against X1	-	23.1***	-	-	-	-	-
LR χ^2^ test against X2	-	-	86.6***	92.4***	3.3	17.3***	152.6***
B. Share of ASFR 40+ in TFR							
Population density	0.66***	0.62***	0.39***	0.29***	0.61***	0.54***	0.23***
SD_c_ Population density		0.29	0.21	0.21	0.29	0.28	0.18
Education							
Share of women with tertiary education			0.43***				0.37***
Economic environment							
GDP/capita (%)				0.22***			0.13*
Share of high-tech sector				0.31***			0.12*
Family and gender norms							
Share of divorced persons (2011)					0.02		-0.01
Ratio of male-to-female employment (age 25-54)					-0.03		0.12**
Population composition							
Share of population age 60+						-0.10*	-0.03
Share of foreign-born female population (age 25-54)						0.04**	0.03*
Intercept	1.75**	0.79*	0.40	0.27	0.74*	0.89**	0.49
SD_c_ intercept	3.30	1.77	1.62	1.57	1.75	1.52	1.46
SD residual	0.68	0.64	0.56	0.57	0.64	0.64	0.53
AIC	704	675	596	604	678	667	573
BIC	719	693	617	629	703	692	617
LR χ^2^ test against X1	-	31.1***	-	-	-	-	-
LR χ^2^ test against X2	-	-	81.4***	75.1***	0.98	12.2**	115.8***

*Note*: *N*_country_ = 28; *N*_NUTS__2_ = 270. b, regression coefficients. SD_c_, random-effect on country level (i.e., variation of coefficient *b* across countries). Abbreviations: AIC, Akaike information criterion; ASFR, age-specific fertility rates; BIC, Bayesian information criterion; GDP, gross domestic product; LR, likelihood ratio; TFR, total fertility rate.

* p ≤ 0.05

** p ≤ 0.01

*** p ≤ 0.001

**** p ≤ 0.10

**Table 3:** Associations between covariates and later fertility on NUTS 2 level in models with population density and only one other covariate (multilevel mixed regression models), standardisation with grand mean and within-country standard.

Models incl. population density and one additional covariate Other covariate	Share of ASFR 35+ in TFR (NUTS 2)			Share of ASFR 40+ in TFR (NUTS 2)		
	Population density		Other covariate	Population density		Other covariate
	b	SD_c_	b	b	SD_c_	b
None	0.55***	0.28	-	0.62***	0.29	-
Education						
Share of women with tertiary education	0.30***	0.19	0.49***	0.39***	0.21	0.43***
Economic environment						
GDP/capita (%)	0.27***	0.22	0.44***	0.38***	0.24	0.36***
Share of high-tech sector	0.26***	0.21	0.49***	0.37***	0.22	0.41***
Family and gender norms						
Share of divorced persons (2011)	0.57***	0.28	-0.08****	0.61***	0.29	0.03
Ratio of male-to-female employment	0.55***	0.28	-0.01	0.61***	0.29	-0.04
Population composition						
Share of population age 60+	0.49***	0.25	-0.15**	0.58***	0.27	-0.10*
Share of foreign-born female population (age 25–54)	0.51***	0.28	0.04***	0.58***	0.29	0.04**

*Note*: *N*_country_ = 28; *N*_NUTS 2_ = 270; *N*_NUTS 3_ = 1328. b, regression coefficients. SD_c_, random-effect on country level (i.e., variation of coefficient *b* across countries).

Abbreviation: GDP, gross domestic product.

* p ≤ 0.05

** p ≤ 0.10;

*** p ≤ 0.001;

**** p ≤ 0.10

## Data Availability

The article combines Eurostat data from different sources that is publicly available. Detailed descriptions and links are presented to the reader in a table in the Supporting Information: Appendix.

## Footnotes

1 In most countries, figures are available for 5-year age groups (10−14,15−19, 20−24, 25−29, 30−34. 35−39, 40−44, 45−49, 50+). For the United Kingdom and Germany, figures begin with the age group ‘below age 20’ and end with the age group ‘age 45+’. Computing agespecific fertility, we assumed 10-year intervals for ‘below age 20’ and ‘age 45+’ and a 5-year interval for ‘age 50+’.

2 If data for 2018 was not available for single regions, we used the chronologically closest available information to fill these gaps (usually, 2017).

3 As categories have been changed in several countries between NUTS 2010 and NUTS 2013 (France, Germany, Greece, Poland, Portugal, Slovenia, United Kingdom), NUTS 2013 and NUTS 2016 (Finland, Germany, Ireland, Netherlands, Poland, United Kingdom) and NUTS 2016 and NUTS 2021 (Croatia, Norway), and given the restricted availability of more detailed subregional data, we imputed the values for NUTS 2016 regions using the NUTS converter of the Joint Research Centre (2023) and based on population size in 2010 (for 2011 census data) or 2018 (share of high-tech sector 2018), respectively. Changes in and of NUTS regions are listed in the Supporting Information: Table A.2.

4 Some countries that have been included in the descriptive analyses had to be excluded due to missing information on context characteristics. The remaining 28 countries are Austria, Belgium, Bulgaria, Croatia, Cyprus, Czechia, Denmark, Estonia, Finland, France, Germany, Greece, Hungary, Italy, Latvia, Lithuania, Luxembourg, Malta, the Netherlands, Norway, Poland, Portugal, Romania, Sweden, Slovakia, Slovenia, Spain and the United Kingdom. Within those countries, some NUTS 2 and NUTS 3 regions had to be omitted from the analyses because of missing context information as well (Finland: 1 NUTS 3 region; France: 5; Greece: 10; Italy: 1; Poland: 2; Portugal: 8; Spain: 2). Most of them are autonomous regions, islands or oversea departments (a limitation for prior research as well; e.g., Campisi et al., 2020).

5 Spatial models with standard weight matrices account for proximity effects from bidirectional spillover. If similarity due to proximity results from effects of urban centres on surrounding areas but similarity of surrounding areas to urban areas is used to explain effects of urban regions in a cross-sectional setting, findings could be misleading. Using information from population density to generate a weight matrix that guarantees larger influences of regions with higher population density, for instance, would further complicate the interpretation of direct and indirect effects of population density. In addition, the correct specification of multilevel models that allow for spatial random effects and spatial random slopes (e.g., by following Bayesian approaches) and its estimation are not straightforward.

6 Unfortunately, the number of NUTS 2 regions has not been sufficient to compute meaningful standard deviations for Croatia, Latvia, Lithuania, Luxembourg and Malta; for NUTS 3 regions for Luxembourg and Malta. In these exceptional cases, the standard deviation of the total sample of regions had to be used.

7 See Footnote 6.
