# Supplementary material for: Explaining the urban–rural gradient in later fertility in Europe
Source: Popul Space Place. Author manuscript; Available in PMC 2024 Jan 11. (PMC7615507; doi:10.1002/psp.2720)
Supplement: Supplementary material [file EMS190342-supplement-Supplementary_material.docx]

**Appendix Tables and Figures**

**Table A.1 Description of variables at NUTS 2 and NUTS 3 level**

| 28 European countries | NUTS 3 level | | NUTS 2 level | | Data description |
| --- | --- | --- | --- | --- | --- |
| Data for 2018 (or 2011),  except Germany (2017) | Mean | SD | Mean | SD | and source |
| Main interest variables |  |  |  |  |  |
| share of ASFR 35+ in TFR (%) | 21.4 | 6.3 | 22.1 | 6.0 | Calculated by authors using information on live births by age group of the mothers [demo_r_fagec3]^1^ and age of female population on 1 Jan [demo_r_pjangrp3]^2^ |
| share of ASFR 40+ in TFR (%) | 4.0 | 1.8 | 4.2 | 1.7 |  |
| population density | 605.6 | 1504.4 | 461.5 | 1248.2 | Persons per square kilometre [demo_r_d3dens]^1^ |
| *Economic environment* |  |  |  |  |  |
| GDP/capita (% of EU average) | 95.0 | 61.2 | 97.3 | 46.2 | Gross domestic product in purchasing power standard per inhabitant in percentage of the EU27 average [nama_10r_3gdp]^3^ |
| share of high-tech sector (%) | - | - | 3.7 | 2.0 | Share of employment in high-technology sectors (high-technology manufacturing and knowledge-intensive high-technology services) [htec_emp_reg2]^4^ |
| *Family and gender norms* |  |  |  |  |  |
| share of divorced persons (2011) | 6.1 | 2.3 | 6.4 | 2.3 | Share of divorced persons and persons whose registered partnership was legally dissolved among total population; calculated by authors based on census data 2011 [cens_11ms_r3]^5^ |
| ratio of male-to-female employment (age 25-54) | - | - | 1.2 | .1 | Calculation by authors using labour force data [lfst_r_lfe2emprtn]^6^ |
| *Population composition* |  |  |  |  |  |
| share of population age 60+ | 27.3 | 4.2 | 26.4 | 3.4 | Calculated by authors using data from 1 Jan 2018 (2017 for Germany) [demo_r_pjangrp3]^2^ |
| share of foreign-born among female population (%) | - | - | 14.8 | 11.0 | Share of foreign-porn among the female population age 25-54; calculated by authors [lfst_r_lfsd2pwc]^7^ |
| *Education* |  |  |  |  |  |
| share of women with tertiary education (%) | - | - | 34.8 | 11.2 | Share of women age 25-64 with tertiary education (ISCED levels 5-8) [edat_lfse_04]^8^ |
| N | 1,328 |  | 270 |  |  |

Note: Data refers to 2018 for all countries except Germany (2017) and all variables except those stemming from 2011 censuses. For eight NUTS 2 regions, figures for share of high-tech sector refer to previous years due to missing values in 2017 or 2018, respectively.

Source: EUROSTAT, retrieved from ^1^ <https://ec.europa.eu/eurostat/web/rural-development/data> (28 Jan 2021),
^2^ <https://ec.europa.eu/eurostat/databrowser/view/DEMO_R_PJANGRP3__custom_665459/default/table?lang=en> (16 Aug 2021),
^3^ <http://appsso.eurostat.ec.europa.eu/nui/show.do?dataset=nama_10r_3gdp> (28 Jan 2021),
^4^ <https://appsso.eurostat.ec.europa.eu/nui/show.do?dataset=htec_emp_reg2&lang=en> (1 Mar 2021),
^5^ <https://ec.europa.eu/eurostat/databrowser/view/CENS_11MS_R3/default/table> (1 Mar 2021),
^6^ <https://appsso.eurostat.ec.europa.eu/nui/show.do?dataset=lfst_r_lfe2emprtn&lang=en> (11 Mar 2021),
^7^ <http://data.europa.eu/88u/dataset/uhwlwjmeersx5oiciwkrg> (27 Jul 2023),
^8^ <https://ec.europa.eu/eurostat/databrowser/view/EDAT_LFSE_04__custom_532852/default/table?lang=en> (4 Feb 2021).

**Table A.2 Information regarding NUTS region changes 2010/2013 and 2013/2016**

| **Old code(s)** | **New code(s)** | **Label** | **Change** | **Additional information** |
| --- | --- | --- | --- | --- |
| **2010** | **2013** |  |  |  |
| DE80B, DE80C, DE802, DE808 | DE80J | Mecklenburgische Seenplatte | new region | DE80J = DE80C + DE80B + DE802 +  parts of DE808 |
| DE80F, DE80I, DE801, DE808 | DE80N | Vorpommern-Greifswald | new region | DE80N = DE801 + DE80F + DE80I +  parts of DE808 |
| DE807, DE809 | DE80K | Landkreis Rostock | new region | DE80K = DE807 + DE809 |
| DE80D, DE80H, DE805 | DE80L | Vorpommern-Rügen | new region | DE80L = DE805 + DE80D + DE80H |
| DE80E, DE806 | DE80M | Nordwestmecklenburg | new region | DE80M = DE806 + DE80E |
| DE80A, DE80G | DE80O | Ludwigslust-Parchim | new region | DE80O = DE80A + DE80G |
| EL131, EL133 | EL531 | Γρεβενά, Κοζάνη | new region | EL531 = EL131 + EL133 |
| EL211, EL214 | EL541 | Άρτα, Πρέβεζα | new region | EL541 = EL211 + EL214 |
| EL141, EL144 | EL611 | Καρδίτσα, Τρίκαλα | new region | EL611 = EL141 + EL144 |
| EL251, EL252 | EL651 | Αργολίδα, Αρκαδία | new region | EL651 = EL251 + EL252 |
| EL254, EL255 | EL653 | Λακωνία, Μεσσηνία | new region | EL653 = EL254 + EL255 |
| EL300 | EL301 | Βόρειος Τομέας Αθηνών | new region | part of EL300 |
| EL300 | EL302 | Δυτικός Τομέας Αθηνών | new region | part of EL300 |
| EL300 | EL303 | Κεντρικός Τομέας Αθηνών | new region | part of EL300 |
| EL300 | EL304 | Νότιος Τομέας Αθηνών | new region | part of EL300 |
| EL300 | EL305 | Ανατολική Αττική | new region | part of EL300 |
| EL300 | EL306 | Δυτική Αττική | new region | part of EL300 |
| EL300 | EL307 | Πειραιάς, Νήσοι | new region | part of EL300 |
| FR91 | FRA1, FRA10 | Guadeloupe | boundary shift | since 2016 code **FRY1, FRY10** |
| - | FRA5, FRA50 | Mayotte | new region | since 2016 code **FRY5, FRY50** |
| PL121, PL122 | PL12B | Ciechanowski | new region | PL12B = part of PL121 + part of PL122 |
| PL121 | PL12C | Płocki | new region | part of PL121 |
| PL122 | PL12D | Ostrołęcki | new region | part of PL122 |
| - | PL12E | Siedlecki | new region |  |
| PL215 | PL218 | Nowosądecki | new region | PL218 = part of PL215 |
| PL215, PL216 | PL219 | Nowotarski | new region | PL219 = part of PL215 + part of PL216 |
| PL216 | PL21A | Oświęcimski | new region | PL21A = part of PL216 |
| PL422 | PL426 | Koszaliński | new region | PL426 = part of PL422 |
| PL422, PL423 | PL427 | Szczecinecko-pyrzycki | new region | PL427 = part of PL422 + part of PL423 |
| PL423, PL425 | PL428 | Szczeciński | new region | PL428 = PL425 + part of PL423 |
| PL521, PL522 | PL523 | Nyski | new region | PL523 = part of PL521 + part of PL522 |
| PL521, PL522 | PL524 | Opolski | new region | PL524 = part of PL521 + part of PL522 |
| PL614, PL615 | PL616 | Grudziądzki | new region | PL616 = part of PL614 + part of PL615 |
| PL615 | PL617 | Inowrocławski | new region | PL617 = part of PL615 |
| PL614 | PL618 | Świecki | new region | PL618 = part of PL614 |
| PL615 | PL619 | Włocławski | new region | PL619 = part of PL615 |
| PL631 | PL636 | Słupski | new region | PL636 = part of PL631 |
| PL631, PL635 | PL637 | Chojnicki | new region | PL637 = part of PL631 + part of PL635 |
| PL635 | PL638 | Starogardzki | new region | PL638 = part of PL635 |
| PT113, PT115 | PT119 | Ave | new region | PT119 = part of PT113+part of PT115 |
| PT114, PT115, PT116 | PT11A | Área Metropolitana do Porto | new region | PT11A = PT116 + part of PTPT114 +  part of PT115 |
| PT115, PT118 | PT11B | Alto Tâmega | new region | PT11B = part of PT115 + part of PT118 |
| PT115 | PT11C | Tâmega e Sousa | new region | PT11C = part of PT115 |
| PT117 | PT11D | Douro | new region | PT11D = part of PT117 |
| PT117, PT118 | PT11E | Terras de Trás-os-Montes | new region | PT11E = part of PT117 + part of PT118 |
| PT161 | PT16D | Região de Aveiro | new region | PT16D = part of PT161 |
| PT161, PT162, PT164 | PT16E | Região de Coimbra | new region | PT16E = part of PT161 + PT162 + part of PT164 |
| PT163, PT164 | PT16F | Região de Leiria | new region | PT16F = PT163 + part of PT164 |
| PT165 | PT16G | Viseu Dão Lafões | new region | PT16G = part of PT165 |
| PT166, PT169 | PT16H | Beira Baixa | new region | PT16H = part of PT166 + PT169 |
| PT166, PT16C | PT16I | Médio Tejo | new region | PT16I = PT16C + part of PT166 |
| PT167, PT168, PT16A | PT16J | Beiras e Serra da Estrela | new region | PT16J = PT167 + PT168 + PT16A |
| PT171, PT172 | PT170 | Área Metropolitana de Lisboa | new region | PT170 = PT171 + PT172 |
| PT182, PT183 | PT186 | Alto Alentejo | new region | PT186 = part of PT182 + part of PT183 |
| PT182, PT183 | PT187 | Alentejo Central | new region | PT187 = part of PT182 + part of PT183 |

| **Old code(s)** | **New code(s)** | **Label** | **Change** | **Additional information** |
| --- | --- | --- | --- | --- |
| **2010** | **2013** |  |  |  |
| SI01 | SI03 | Vzhodna Slovenija | boundary shift |  |
| SI02 | SI04 | Zahodna Slovenija | boundary shift |  |
| SI014, SI016 | SI034 | Savinjska | new region | SI034 = part of SI014 + part of SI016 |
| SI015, SI021 | SI035 | Zasavska | new region | SI035 = SI015 + part of SI021 |
| SI014, SI016 | SI036 | Posavska | new region | SI036 = part of SI014 + part of SI016 |
| SI021 | SI041 | Osrednjeslovenska | new region | SI041 = part of SI021 |
| UKD31 | UKD33, UKD34, UKD34 | Greater Manchester South | split | UKD31 = UKD33 + UKD34 + UKD35 |
| UKD32 | UKD36, UKD37 | Greater Manchester North | split | UKD32 = UKD36 + UKD37 |
| UKD43 | UKD44, UKD45, UKD46, UKD47 | Lancashire CC | split | UKD32 = UKD44 + UKD45 + UKD46 + UKD47 |
| UKH13 | UKH15. UKH16, UKH17 | Norfolk | split | UKH13 = UKH15 + UKH16 + UKH17 |
| UKH33 | UKH34, UKH35, UKH36, UKH37 | Essex CC | split | UKH33 = UKH34 + UKH35 + UKH36 + UKH37 |
| UKI1 | UKI3, UKI3 | Inner London | split | UKI1 = UKI3 + UKI4 |
| UKI11 | UKI31, UKI32, UKI33, UKI34 | Inner London - West | split | UKI11 = UKI31 + UKI32 + UKI33 + UKI34 |
| UKI12 | UKI41, UKI42, UKI43, UKI44, UKI45 | Inner London - East | split | UKI12 = UKI41 + UKI42 + UKI43 + UKI44 + UKI45 |
| UKI2 | UKI5, UKI6, UKI7 | Outer London | split | UKI2 = UKI5 + UKI6 + UKI7 |
| UKI21 | UKI51, UKI52, UKI53, UKI54 | Outer London - East and North East | split | UKI21 = UKI51 + UKI52 + UKI53 + UKI54 |
| UKI22 | UKI61, UKI62, UKI63 | Outer London - South | split | UKI22 = UKI61 + UKI62 + UKI63 |
| UKI23 | UKI71, UKI72, UKI73, UKI74, UKI75 | Outer London - West and North West | split | UKI23 = UKI71 + UKI72 + UKI73 + UKI74 + UKI75 |
| UKJ23 | UKJ25, UKJ26 | Surrey | split | UKJ23 = UKJ25 + UKJ26 |
| UKJ24 | UKJ27, UKJ28 | West Sussex | split | UKJ24 = UKJ27 + UKJ28 |
| UKJ33 | UKJ35, UKJ36, UKJ37 | Hampshire CC | split | UKJ33 = UKJ35 + UKJ36 + UKJ37 |
| UKJ42 | UKJ43, UKJ44, UKJ45, UKJ46 | Kent CC | split | UKJ42 = UKJ43 + UKJ44 + UKJ45 + UKJ46 |
| **Old code(s)** | **New code(s)** | **Label** | **Change** | **Additional information** |
| **2013** | **2016** |  |  |  |
| DE915, DE919 | DE91C | Göttingen | new region | DE91C = DE915 + DE919 |
| DEB16 | DEB1C | Cochem-Zell | boundary shift |  |
| DEB19 | DEB1D | Rhein-Hunsrück-Kreis | boundary shift |  |
| FI1D4 | FI1D8 | Kainuu | boundary shift |  |
| FI1D6 | FI1D9 | Pohjois-Pohjanmaa | boundary shift |  |
| IE01, IE02 | IE04 | Northern and Western | new region |  |
| IE011 | IE041 | Border | boundary shift |  |
| IE01, IE02 | IE05 | Southern | new region | IE05 = IE023 + IE024 + IE025 |
| IE023 | IE051 | Mid-West | boundary shift |  |
| IE024 | IE052 | South-East | boundary shift |  |
| IE01, IE02 | IE06 | Eastern and Midland | new region |  |
| IE022 | IE062 | Mid-East | boundary shift |  |
| - | LT02 | Vidurio ir vakarų Lietuvos regionas | new region | LT02 = LT00 - LT00A |
| NL121 | NL124 | Noord-Friesland | boundary shift |  |
| NL122 | NL125 | Zuidwest-Friesland | boundary shift |  |
| NL123 | NL126 | Zuidoost-Friesland | boundary shift |  |
| NL322 | NL328 | Alkmaar en omgeving | boundary shift |  |
| NL326 | NL329 | Groot-Amsterdam | boundary shift |  |
| NL338 | NL33B | Oost-Zuid-Holland | boundary shift |  |
| NL339 | NL33C | Groot-Rijnmond | boundary shift |  |
| PL12 | PL91 | Warszawski stołeczny | new region | PL91 = PL127 + PL129 + PL12A - new PL926 |
| PL12 | PL92 | Mazowiecki regionalny | new region | PL92 = PL128 + PL12B + PL12C + PL12D + PL12E + new PL926 |
| PL129 | PL912 | Warszawski wschodni | boundary shift |  |
| PL12A | PL913 | Warszawski zachodni | boundary shift | PL913 = PL12A - new PL926 |
| PL12E | PL925 | Siedlecki | boundary shift |  |
| - | PL926 | Żyrardowski | new region |  |

| **Old code(s)** | **New code(s)** | **Label** | **Change** | **Additional information** |
| --- | --- | --- | --- | --- |
| **2013** | **2016** |  |  |  |
| UKM2 | UKM7 | Eastern Scotland | boundary shift | UKM7 = UKM2 - UKM24 |
| UKM3 | UKM8 | West Central Scotland | new region | UKM8 = UKM31 + UKM34 + UKM35 + UKM36 |
| UKM3 | UKM9 | Southern Scotland | new region | UKM9 = UKM24 + UKM32 + UKM33 + UKM37 + UKM38 |
| UKN01 | UKN06 | Belfast | boundary shift |  |
| UKN03, UKN05 | UKN07 | Armagh City, Banbridge and Craigavon | new region | part of UKN03 + part of UKN05 |
| UKN03, UKN05 | UKN08 | Newry, Mourne and Down | new region | part of UKN03 + part of UKN05 |
| UKN03 | UKN09 | Ards and North Down | new region | part of UKN03 |
| UKN04 | UKN10 | Derry City and Strabane | new region | part of UKN04 |
| UKN05 | UKN11 | Mid Ulster | new region | part of UKN05 |
| UKN04 | UKN12 | Causeway Coast and Glens | new region | part of UKN04 |
| UKN03, UKN02 | UKN13 | Antrim and Newtownabbey | new region | part of UKN02 + part of UKN03 |
| UKN02 | UKN14 | Lisburn and Castlereagh | new region | part of UKN02 |
| UKN03, UKN02 | UKN15 | Mid and East Antrim | new region | part of UKN02 + part of UKN03 |
| UKN05 | UKN16 | Fermanagh and Omagh | new region | part of UKN05 |
| **Old code(s)** | **New code(s)** | **Label** | **Change** | **Additional information** |
| **2016** | **2021** |  |  |  |
| HR04 | HR05 | Grad Zagreb | new region | HR05 = HR041 |
| HR04 | HR06 | Sjeverna Hrvatska | new region | HR06 = HR042 + HR043 +HR044 + HR045 + HR046 |
| HR04 | HR02 | Panonska Hrvatska | new region | HR02 = HR047 + HR048 +HR049 + HR04A + HR04B + HR04C + HR04D + HR04E |
| NO01 |  | Oslo og Akerhus | discontinued |  |
| NO03 |  | Sør-Østlandet | discontinued |  |
| NO04 |  | Agder og Rogaland | discontinued |  |
|  | NO08 | Oslo og Viken | new region |  |
|  | NO09 | Agder og Sør-Østlandet | new region |  |
| NO05 | NO0A | Vestlandet | boundary shift |  |
|  | NO0B | Jan Mayen and Svalbard | new region |  |

Source: EUROSTAT, retrieved from <https://ec.europa.eu/eurostat/web/nuts/history> (26 Jul 2023).

**Table A.3: Associations between population density and later fertility on NUTS 2 level (multilevel mixed regression models), standardised using grand mean and standard deviation across all NUTS 2 regions**

| model | Y1 |  | Y2 |  | Y3 |  | Y4 |  | Y5 |  | Y6 |  | Y7 | | |  |
| --- | --- | --- | --- | --- | --- | --- | --- | --- | --- | --- | --- | --- | --- | --- | --- | --- |
| **A. Share of ASFR 35+ in TFR** | | | | | | | | | | | | | | | | |
| Population density (b) | .42 | *** | 1.13 | *** | .27 | *** | .20 | *** | 1.20 | *** | .73 | *** | .09 | ** | | |
| *SD_c_ Population density* |  |  | *.82* |  | *.00* |  | *.00* |  | *.89* |  | *.56* |  | *.00* |  | | |
| **B. Share of ASFR 40+ in TFR** | | | | | | | | | | | | | | | | |
| Population density (b) | .53 | *** | 1.18 | *** | .40 | *** | .34 | *** | 1.17 | *** | .86 | *** | .24 | *** | | |
| *SD_c_ Population density* |  |  | *.78* |  | *.00* |  | *.00* |  | *.76* |  | *.57* |  | *.00* |  | | |
| Included in model: |  |  |  |  |  |  |  |  |  |  |  |  |  | |  | |
| Education | - |  | - |  | incl. |  | - |  | - |  | - |  | incl. | |  | |
| Economic environment | - |  | - |  | - |  | incl. |  | - |  | - |  | incl. | |  | |
| Family and gender norms | - |  | - |  | - |  | - |  | incl. |  | - |  | incl. | |  | |
| Population composition | - |  | - |  | - |  | - |  | - |  | incl. |  | incl. | |  | |

Note: N _country_ = 28; N _NUTS 2_ = 270. *** p ≤ .001, ** p ≤ .01. Economic environment: GDP/capita (%), share of high-tech sector; family and gender norms: share of divorced persons (2011), share of couples with four or more children (2011); ratio of male-to-female employment (employment shares, age 25-54); population composition: share of population age 60+, share of foreign-born female population age 25-54; education: share of women with tertiary education. b indicates regression coefficients. SD_c_ indicates random-effect on country level (i.e. variation of coefficient b across countries).

**Table A.4: Regression coefficients of population density in different regression model specifications, standardisation with grand mean and within-country standard deviation**

| Model | X2 or X1 | | X3 |  | | X4 |  | X5 |  | | X6 |  | X7 |  |
| --- | --- | --- | --- | --- | --- | --- | --- | --- | --- | --- | --- | --- | --- | --- |
| **A. Share of ASFR 35+ in TFR** | | | | | | | | | | | | | | |
| Mixed multilevel regression with random effect of pop. density | .55 | *** | .30 | *** | | .16 | * | .58 | *** | | .45 | *** | .12 | * |
| Mixed multilevel regression without random effect of pop. | .55 | *** | .27 | *** | | .15 | ** | .58 | *** | | .42 | *** | .08 | ^(^*^)^ |
| Regression with country fixed effects and adj. standard errors | .66 | *** | .34 | *** | | .22 | ** | .68 | *** | | .47 | *** | .13 | * |
| Difference in coefficients compared to X2 (Chi² test) |  |  | 54.48 | *** | | 54.38 | *** | 1.26 |  | 4.77 | | * | 48.00 | *** |
| **B. Share of ASFR 40+ in TFR** | | | | | | | | | | | | | | |
| Mixed multilevel regression with random effect of pop. density | .62 | *** | .39 | *** | | .29 | *** | .61 | *** | | .54 | *** | .23 | *** |
| Mixed multilevel regression without random effect of pop. | .66 | *** | .37 | *** | | .28 | *** | .66 | *** | | .56 | *** | .19 | *** |
| Regression with country fixed effects and adj. standard errors | .74 | *** | .46 | *** | | .39 | *** | .74 | *** | | .56 | *** | .28 | *** |
| Difference in coefficients compared to X2 (Chi² test) |  |  | 66.73 | ** | 126.90 | | *** | .00 |  | | 11.30 | *** | 61.39 | *** |
| Included in model: |  |  |  |  | |  |  |  |  |  | |  |  |  |
| Education | - |  | incl. |  | |  |  | - |  | - | |  | incl. |  |
| Economic environment | - |  | - |  | | incl. |  | - |  | - | |  | incl. |  |
| Family and gender norms | - |  | - |  | | - |  | incl. |  | - | |  | incl. |  |
| Population composition | - |  | - |  | | - |  | - |  | incl. | |  | incl. |  |

Note: N _country_ = 28; N _NUTS 2_ = 270. *** p ≤ .001, ** p ≤ .01, * p ≤ .05, ^(^*^)^ p ≤ .10.

**Table A.5: Associations between covariates and later fertility on NUTS 3 and NUTS 2 level (multilevel mixed regression models), standardisation with grand mean and within-country standard deviation**

| Share of ASFR in TFR (NUTS 3) | 35+ |  | 35+ |  | 35+ |  | 40+ |  | 40+ |  | 40+ |  |
| --- | --- | --- | --- | --- | --- | --- | --- | --- | --- | --- | --- | --- |
| model | M2 |  | M3 |  | M4 |  | M2 |  | M3 |  | M4 |  |
|  | b |  | b |  | b |  | b |  | b |  | b |  |
| NUTS 3 level covariates |  |  |  |  |  |  |  |  |  |  |  |  |
| Population density | .53 | *** | .26 | *** | .24 | *** | .53 | *** | .30 | *** | .28 | *** |
| GDP/capita (%) |  |  | .28 | *** | .25 | *** |  |  | .21 | *** | .17 | *** |
| share of divorced persons (2011) |  |  | .07 |  | .06 |  |  |  | .12 | ** | .11 | * |
| share of population age 60+ |  |  | -.12 | ^(^*^)^ | -.11 | ^(^*^)^ |  |  | -.12 | * | -.12 | * |
| NUTS 2 level covariates |  |  |  |  |  |  |  |  |  |  |  |  |
| share of women with tertiary education |  |  |  |  | .24 | *** |  |  |  |  | .25 | *** |
| share of high-tech sector |  |  |  |  | -.05 |  |  |  |  |  | -.04 |  |
| ratio of male-to-female employment |  |  |  |  | .03 |  |  |  |  |  | .02 |  |
| share of foreign-born female population (age 25-54) |  |  |  |  | .02 | * |  |  |  |  | .02 | ^(^*^)^ |
| Intercept | .78 |  | .23 |  | .44 |  | .71 |  | .27 |  | .44 |  |
| Random effects at country level |  |  |  |  |  |  |  |  |  |  |  |  |
| SD_c_ Population density | .18 |  | .09 |  | .06 |  | .21 |  | .14 |  | .11 |  |
| SD_c_ GDP/capita (%) |  |  | .12 |  | .11 |  |  |  | .09 |  | .08 |  |
| SD_c_ share of divorced persons (2011) |  |  | .23 |  | .24 |  |  |  | .16 |  | .17 |  |
| SD_c_ share of population age 60+ |  |  | .27 |  | .26 |  |  |  | .23 |  | .22 |  |
| SD_c_ intercept | 2.20 |  | 1.65 |  | 1.48 |  | 1.94 |  | 1.56 |  | 1.44 |  |
| Random effects at NUTS 2 level |  |  |  |  |  |  |  |  |  |  |  |  |
| SD_N2_ intercept | .47 |  | .37 |  | .32 |  | .42 |  | .36 |  | .31 |  |
| SD_N2_ residual | .66 |  | .58 |  | .58 |  | .67 |  | .61 |  | .61 |  |

Note: N _country_ = 28; N _NUTS 2_ = 270; N _NUTS 3_ = 1,328. *** p ≤ .001, ** p ≤ .01, * p ≤ .05, ^(^*^)^ p ≤ .10. b indicates regression coefficients. SD_c_ and SD_N2_ indicate random-effects on country level and NUTS 2 level, respectively (variation of coefficient b across countries or NUTS 2 regions, respectively).

**Table A.6: Associations between covariates and later fertility on NUTS 3 and NUTS 2 level in models including population density and only one other covariate (multilevel mixed regression models), standardisation with grand mean and within-country standard deviation**

| **Models incl. population density and one additional covariate** | Share of ASFR 35+  in TFR (NUTS 3) | Share of ASFR 40+  in TFR (NUTS 3) |
| --- | --- | --- |

|  | Population density | | | Other  covariate | | | Population density | | | Other  covariate | | |
| --- | --- | --- | --- | --- | --- | --- | --- | --- | --- | --- | --- | --- |
|  |  |  |  |  |  |  |  |  |  |  |  |  |
| Other covariate | b |  | SD_c_ | b |  | SD_c_ | b |  | SD_c_ | b |  | SD_c_ |
| none | .53 | *** | .18 | - |  | - | .53 | *** | .21 |  | - | - |
| NUTS 3 level |  |  |  |  |  |  |  |  |  |  |  |  |
| GDP/capita (%) | .31 | *** | *.15* | .35 | *** | *.19* | .35 | *** | *.19* | .29 | *** | *.14* |
| share of divorced persons (2011) | .49 | *** | *.18* | .06 |  | *.20* | .49 | *** | *.21* | .10 | * | *.13* |
| share of population age 60+ | .46 | *** | *.16* | -.17 | ** | *.24* | .48 | *** | *.18* | -.14 | ** | *.21* |
| NUTS 2 level |  |  |  |  |  |  |  |  |  |  |  |  |
| share of women with tertiary education | .47 | *** | *.17* | .26 | *** | - | .46 | *** | *.19* | .27 | *** | - |
| share of high-tech sector | .47 | *** | *.18* | .22 | *** | - | .47 | *** | *.21* | .22 | *** | - |
| ratio of male-to-female employment (age 25-54) | .53 | *** | *.18* | .02 |  | - | .53 | *** | *.21* | -.03 |  | - |
| share of foreign-born female population (age 25-54) | .50 | *** | *.18* | .05 | *** | - | .51 | *** | *.21* | .05 | *** | - |

Note: N _country_ = 28; N _NUTS 2_ = 270; N _NUTS 3_ = 1,328. *** p ≤ .001, ** p ≤ .01, * p ≤ .05. b indicates regression coefficients. SD_c_ indicates random-effect on country level (i.e. variation of coefficient b across countries).

**Table A.7: Spatial dependence in multilevel regression models (Moran’s I)**

| **A. NUTS 3 level** | **35+** | | **40+** |  |
| --- | --- | --- | --- | --- |
| Population density | .81 | *** | .75 | *** |
| Residuals M1 | .01 |  | .02 |  |
| Residuals M2 | .02 |  | .03 |  |
| Residuals M3 | .02 |  | .01 |  |
| Residuals M4 | .02 |  | .02 |  |

| **B. NUTS 2 level** | **35+** | | **40+** |  |
| --- | --- | --- | --- | --- |
| Population density | .80 | *** | .75 | *** |
| Residuals X1 | .20 | *** | .13 | *** |
| Residuals X2 | .22 | *** | .13 | *** |
| Residuals X3 | .30 | *** | .15 | *** |
| Residuals X4 | .25 | *** | .15 | *** |
| Residuals X5 | .21 | *** | .14 | *** |
| Residuals X6 | .20 | *** | .12 | *** |
| Residuals X7 | .18 | *** | .11 | ** |

Note: N _country_ = 28; N _NUTS 2_ = 270; N _NUTS 3_ = 1,328. *** p ≤ .001, ** p ≤ .01.

**Table A.8: Comparison of regression coefficients of population density including results from spatial models based on contiguity matrix, standardisation with grand mean and within-country standard deviation**

| **A. Share of ASFR 35+ in TFR, NUTS 3 level** | | **M2 or M1** | | | **M4** |  |
| --- | --- | --- | --- | --- | --- | --- |
| Mixed multilevel regression with random effect of population density  (and, in M4, other NUTS 3 covariates) | | | .53 | *** | .24 | *** |
| Mixed multilevel regression without random effect of population density | | | .52 | *** | .29 | *** |
| Regression with country fixed effects and adj. standard errors | | | .56 | *** | .30 | *** |
| Spatial model (SAR-SAR) with country effects | direct | | .52 | *** | .28 | *** |
|  | indirect | | .08 | ** | .03 | * |
|  | total | | .60 | *** | .31 | *** |
| Spatial model (SAR-SAR) with country effects  and additional spatial lag of population density | direct | | .52 | *** | .28 | *** |
|  | indirect | | .07 | * | .02 |  |
|  | total | | .59 | *** | .30 | *** |

| **B. Share of ASFR 35+ in TFR, NUTS 2 level** | | **X2 or X1** | | **X7** |  |
| --- | --- | --- | --- | --- | --- |
| Mixed multilevel regression with random effect of population density | | .55 | *** | .12 | * |
| Mixed multilevel regression without random effect of population density | | .55 | *** | .08 | ^(^*^)^ |
| Regression with country fixed effects and adj. standard errors | | .66 | *** | .13 | * |
| Spatial model (SAR-SAR) with country effects | direct | .62 | *** | .08 |  |
|  | indirect | .14 | *** | .01 |  |
|  | total | .76 | *** | .09 |  |
| Spatial model (SAR-SAR) with country effects  and additional spatial lag of population density | direct | .60 | *** | .09 |  |
|  | indirect | .20 | *** | .03 |  |
|  | total | .80 | *** | .12 | ^(^*^)^ |

Note: N _country_ = 28; N _NUTS 2_ = 270; N _NUTS 3_ = 1,328. *** p ≤ .001, ** p ≤ .01, * p ≤ .05, ^(^*^)^ p ≤ .10.

**Figure A.1: Share of ASFR 35+ in TFR by NUTS 3 region, deviation from national average (%)**

**
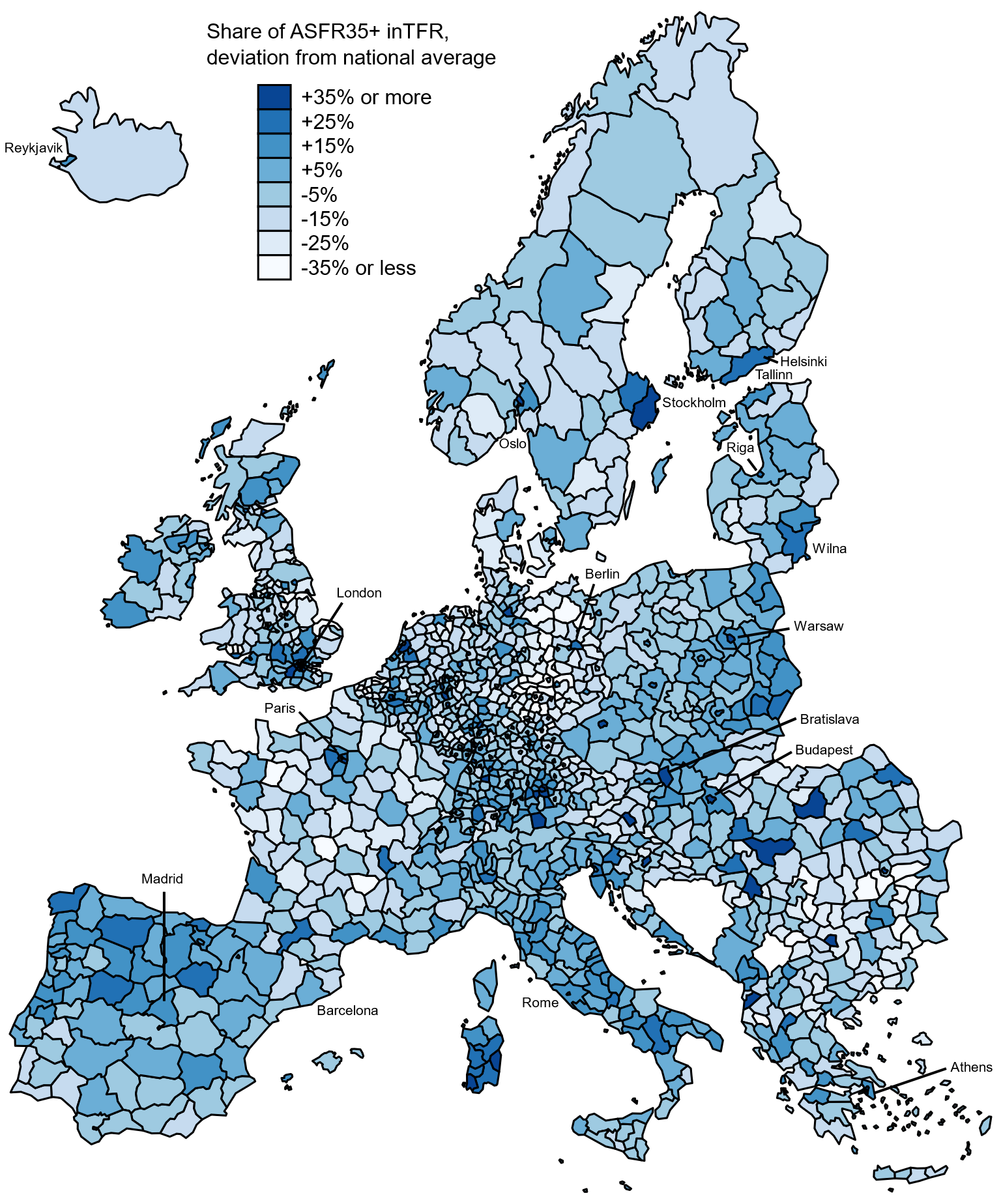
**

Source: Eurostat (2021); own figure.

**Figure A.2: Share of ASFR in TFR by NUTS 2 region, deviation from national average (%)**


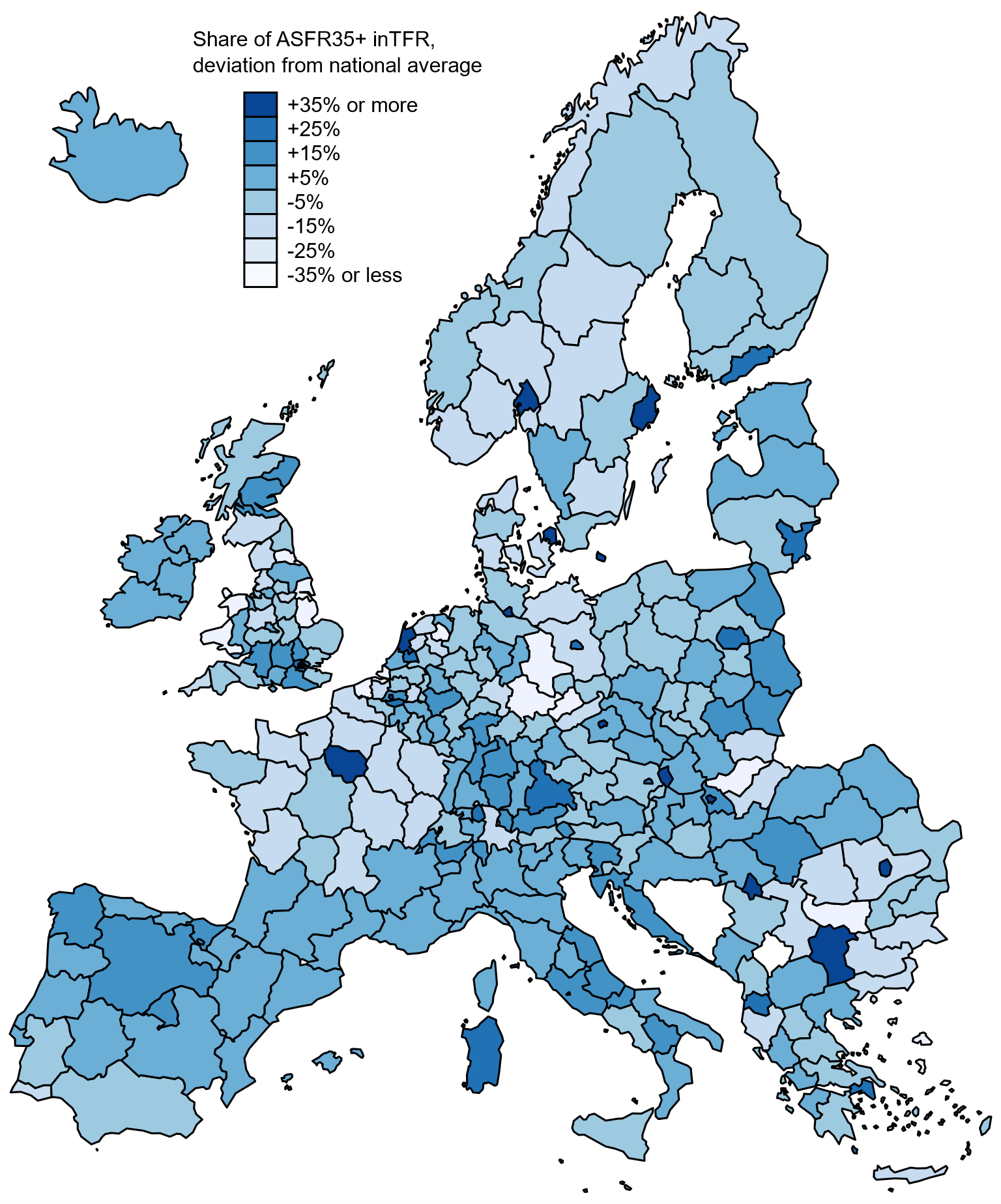

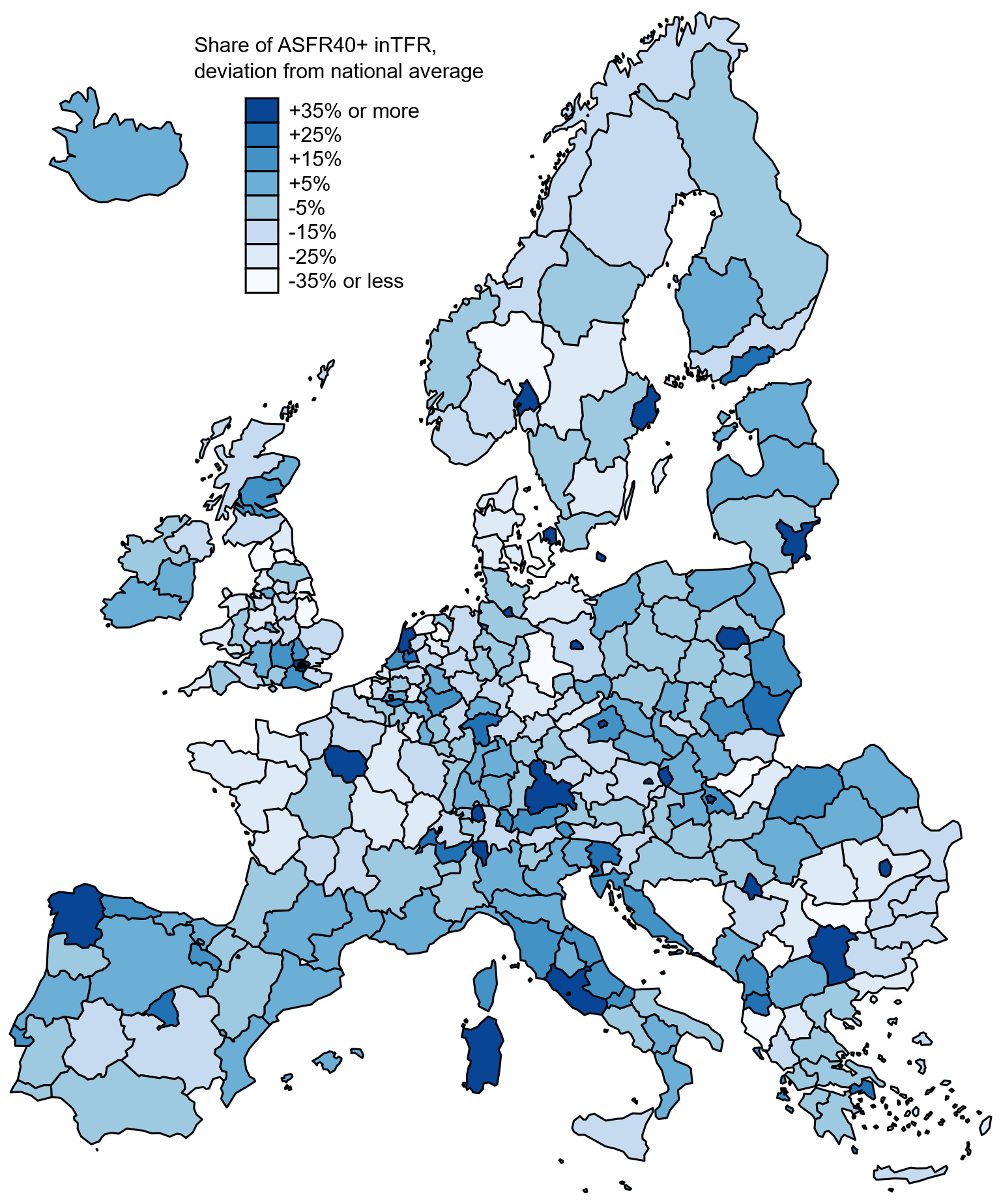


Source: Eurostat (2021); own figures.
